# Using patient-reported outcome measures for primary percutaneous coronary intervention

**DOI:** 10.1136/openhrt-2018-000920

**Published:** 2019-02-16

**Authors:** Esther Kwong, Jenny Neuburger, Steffen Erhard Petersen, Nick Black

**Affiliations:** 1 Department of Health Services Research and Policy, Faculty of Public Health and Policy, London School of Hygiene and Tropical Medicine, London, UK; 2 William Harvey Research Institute, Queen Mary University of London, London, UK; 3 Barts Heart Centre, Barts Health NHS Trust, London, UK

**Keywords:** patient reported outcome measures, health status, health-related quality of life, retrospective, response rate, feasibility, emergency admissions, STEMI, AMI

## Abstract

**Introduction:**

Routine measurement of the outcome of myocardial infarction is usually limited to immediate morbidity and mortality. Our aim was to determine the response to patient-reported outcome measures (PROMs) 3 months later, identify response bias and explore the feasibility of comparing outcome with their recalled view of their prior health state.

**Methods:**

Patients admitted with ST-segment-elevation myocardial infarction (STEMI) to five percutaneous coronary intervention centres were invited to complete a retrospective questionnaire containing the EQ-5D-3L and short form Seattle Angina Questionnaire (SAQ-7). Response rate for a 3-month mailed follow-up questionnaire and potential response biases were assessed. Patients’ outcomes were compared with their baseline using χ^2^ and paired t-test to assess for differences.

**Results:**

Of 392 patients contacted, 260 (66.3%) responded. Responders were more likely to be older, female, more affluent and have a higher EQ-5D at baseline. Three months after surgery, patients’ SAQ-7 and angina symptom subscale returned to their baseline score. The physical limitation subscale score was worse than at baseline (79.9 vs 73.2, p=0.002), whereas the quality-of-life subscale was better (66.6 vs 73.9; p<0.001). The EQ-5D-3L index score was similar at 3 months to baseline (0.82 vs 0.79). Evidence of bias arising from responders being in better general health at baseline needs further investigation and, if confirmed, needs to be taken into account in interpreting PROMs data.

**Conclusion:**

It is feasible to use PROMs routinely to assess the impact of emergency admissions of patients with STEMI. A larger demonstration project with more sites is needed to confirm these findings.

**Key questions**

**What is already known about this subject?**

- While there have been improvements in the management of cardiovascular disease, significant variation still exists in survival following acute myocardial infarction (AMI) between hospitals within England.
- Morbidity and mortality outcomes can be supplemented by patient-reported outcome measures (PROMs), but have not been used widely in routine care.
- The feasibility of recruiting patients with AMI to recall their pre-admission health status has been demonstrated, their likelihood of responding to a postdischarge mailed PROM questionnaire at 3 months is unknown.

**What does this study add?**

- PROMs can be successfully collected in patients 3 months after STEMI with a response rate of 66.3% using mailed follow-up.
- Most patients regained their prior level of cardiac health as measured by the short form Seattle Angina Questionnaire. The physical limitation subscale score was worse than at baseline, whereas the quality-of-life subscale was better.

**How might it impact on clinical practice?**

- PROMs offer the opportunity for routinely assessing the impact of treatment from the patient’s perspective.
- Meaningful comparisons of hospitals based on PROMs could be undertaken to supplement clinical measures such as mortality, morbidity and complications.

## Introduction

Despite the number of emergency admissions to hospital increasing and concern about variations in outcomes between providers,^1 2^ no attempt has been made to use patient-reported outcome measures (PROMs) to determine patients’ perception of their change in health status. In England, emergencies account for about 40% of all hospital admissions, with the number of admissions having increased by 47% over the last 15 years.^3^ Two-thirds of hospital beds are occupied by emergencies and the cost to the National Health Service (NHS) is approximately £12.5 billion annually.^4^

Measuring the quality of healthcare is paramount for all health systems. PROMs is one of the ways to measure effectiveness and to determine the benefit of resources spent.^5 6^ PROMs are self-reported questionnaires designed to be completed by patients to capture their health at specific points in time to detect a health change over a period. They are multidimensional measures which may cover symptoms, functional status or health-related quality of life. Health status and quality of life are outcomes that are highly relevant and important to patients alongside traditional clinical outcomes and survival.^6 7^

While there have been improvements in the management of cardiovascular disease, significant variation still exists in survival following acute myocardial infarction (AMI) between hospitals within England.^8^ However, nothing is known about whether PROMs of survivors also vary between healthcare providers in England as routine assessment is limited to clinical outcomes (mortality and morbidity). Although there have been no attempts in England to routinely capture patients’ recovery using PROMs, they have been used in clinical trials. However, the extent to which such outcomes reflect those obtained in routine clinical care is unclear. There have been attempts to collect longer-term outcomes of patients with AMI in the USA, but whether those results are transferable to the English NHS is unclear.^9–12^

If the aim of healthcare is to restore a patient to his or her full potential, we need to be able to compare patients’ outcomes with their health status before the sudden and unexpected event that leads to the emergency admission. To determine the feasibility of employing PROMs in emergency NHS admissions, an exploratory feasibility study was conducted in patients admitted with ST-segment-elevation myocardial infarction (STEMI).^13^ Success of recruiting patients soon after admission and of obtaining their recollected state of health prior to their admission to provide a baseline assessment has been reported.^13–15^

In this paper, we report on the follow-up response rate for patients who, following an emergency admission, were confirmed to have suffered a STEMI, meeting the PPCI assessment checklist for inclusion and who underwent emergency (primary) percutaneous coronary intervention (PCI). Secondary objectives were to quantify any response bias as regards sociodemographic characteristics, comorbidity and health status and determine its potential impact on outcome assessment. Being an initial feasibility study, it was not powered sufficiently to make meaningful comparisons between participating centres.

## Methods

### Site and patient recruitment

A multisite study was carried out to ensure there would be variation in the administration of patient recruitment and data collection. This would provide insights into the relative merits of recruiting in different settings and with different personnel involved.^16^ For practical reasons, the study was confined to one region of England (North Thames). Five primary angioplasty centres were invited through the National Institute for Health Research (NIHR) Collaborations for Leadership in Applied Health Research and Care partnership network, and all agreed to participate.

Patients admitted with STEMI to the five centres who were alive at discharge were eligible for inclusion unless they were not literate in English, judged not to have sufficient cognitive ability or were not residents in the UK.

Patients were invited to participate soon after their primary PCI and as close to the discharge date as possible to ensure the immediate effects of the intervention were minimised. Clinical staff explained the study to patients, provided written information and obtained written consent. Questionnaires recalling their pre-admission baseline health status were completed by patients without assistance from staff or family except when they were impeded by physical disability or sensory impairment.

Full details of the study methods and feasibility of recruitment have been described elsewhere.^13^

Patients were sent a follow-up questionnaire by mail from the London School of Hygiene and Tropical Medicine 12 weeks after their admission to hospital. Patient vital status was checked against the Personal Demographics Service at NHS Digital prior to sending a follow-up questionnaire. Non-responders after 2 weeks were sent a reminder questionnaire.

### Questionnaires

The questionnaires completed during the admission included demographic information, self-reported comorbidities, a disease-specific PROM and a generic PROM. Patients were asked to recall how they were 1 month before their admission.

The disease-specific PROM used was the short form Seattle Angina Questionnaire (SAQ-7 UK version). This is a seven-item health status measure for patients with coronary artery disease that has well-established validity, reliability, sensitivity to clinical change, and prognostic value.^17–19^ Scores range from 0 to 100, where higher scores indicate fewer symptoms and higher health-related quality of life. SAQ-7 has good domain coverage (symptom burden, functional status and quality of life), psychometric properties (validity, sensitivity), feasibility to implement (questionnaire length, language availability and cost to implement) and clinical interpretability (knowledge of how to interpret scores in a clinically meaningful way).^20^ It assesses five dimensions: exertional capacity, angina stability, angina frequency, treatment satisfaction and disease perception. Three subscales can be derived: physical limitation; angina symptoms; quality of life (SAQ-QoL). The summary scale and the three subscales extend from 0 (worst possible health state) to 100 (best possible health state). The SAQ-7 has been previously validated and applied in patients with acute coronary syndrome.^17 18^

The generic PROM used was the EQ-5D-3L which has five items: mobility, usual activities, personal care, pain/discomfort and anxiety/depression. It takes up to 5 min to complete.^21^ For each item, the patient chooses from three possible responses indicating the level of their function. A multi-attribute utility score where death and perfect health are represented by 0 and 1 is calculated.^22^ Scores less than 0 are considered worse than death and 1 is the maximum score possible. The EQ-5D-3L was used rather than the EQ-5D-5L as the former is still the version used in the National PROMs Programme in England.

### Analysis

Participating patients’ characteristics were summarised using means and SDs for continuous variables or percentages for categorical variables. Response rates were calculated and reported for patients grouped by age, sex, living arrangements, socioeconomic status (SES), baseline SAQ-7 scores and baseline EQ-5D-3L scores. SES was measured using the English Index of Multiple Deprivation (IMD) based on patients’ residential postcodes^23^ with patients assigned to quintiles of the national ranking of IMD scores.

SAQ-7 scores and subscales were calculated according to scoring instructions of the questionnaire developers whereby partial responses were included where possible. Furthermore, individuals with non-responses to two or more items in a subscale did not contribute to the calculation of the component score as per scoring instructions provided by the developers of the SAQ-7^17^

The likelihood of responding according to several patient characteristics (age, sex, SES, comorbidities, baseline SAQ-7 and EQ-5D-3L) was calculated. This allowed the likely impact of non-response on the observed change in SAQ-7 and EQ-5D-3L to be estimated for the patient characteristics shown have a statistically significant non-response association, based on the assumption that non-responders would have reported similar PROM changes as responders.

Patients’ outcomes at 3 months were compared with their baseline using χ^2^ and paired t-test to assess evidence of change in health status. Change scores, with the 95% CIs, were also used to describe reasonable limits on the extent of any change in order to assess whether the results were consistent with recovery to baseline (no change or an improvement in scores).

## Results

### Response rates

A total of 396 patients were recruited and completed questionnaires (Q1) recalling their health state 1 month earlier (online supplementary appendix 1). Of these, four (1%) died during the follow-up period. Of the 392 survivors, 260 patients (66.3%) responded to the follow-up PROM questionnaire (QF), 216 responded to the first request and 44 after one reminder.

10.1136/openhrt-2018-000920.supp1 Supplementary data

The mean time between completing the baseline and the follow-up questionnaire was 89 (SD 17) days and between admission and follow-up questionnaire, 92 days.

### Response bias

**Figure 1:**
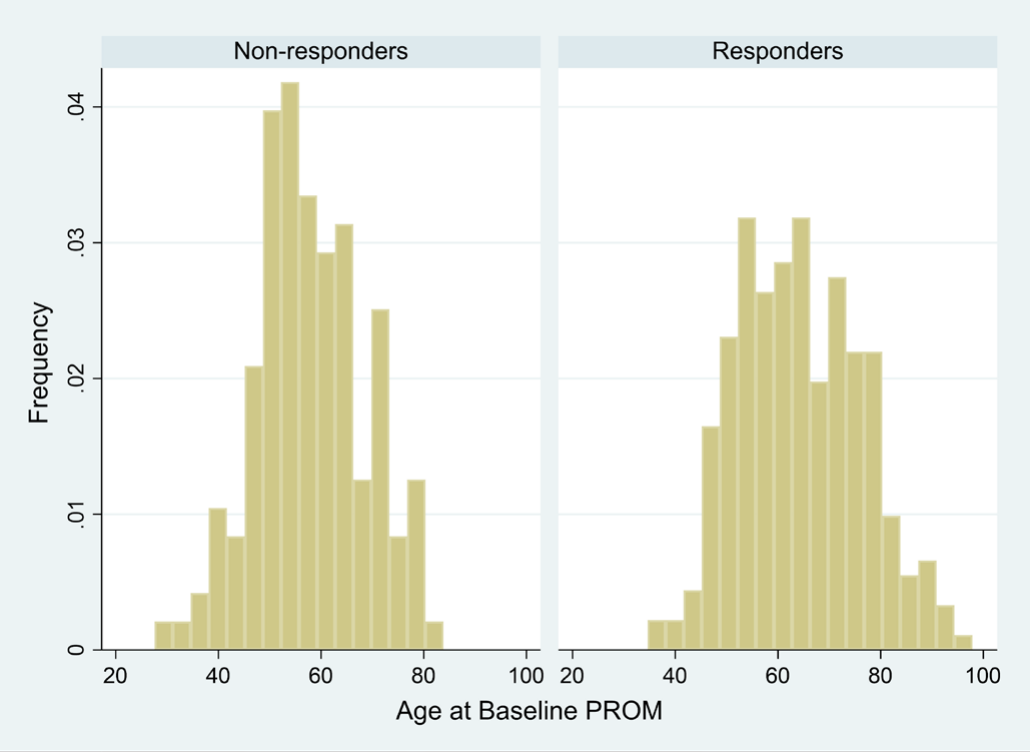
Age distribution of responders and non-responders. PROM, patient-reported outcome measure.

Responders and non-responders were similar as regards comorbidities, living arrangements and disease-specific PROM score (SAQ-7) (table 1). Responders differed from non-responders in other ways: they were older (mean age 64.3 SD 12; range 35–94 vs 57.1, SD 10; range 28–79, p<0.0001) (figure 1), more likely to be women, more likely to come from more affluent SES and have a higher generic PROM score (EQ-5D-3L) at baseline.

**Table 1:** Characteristics of responders (n=260) compared with non-responders (n=132)

Patient characteristics	Overall	Responders	Non-responders	P value
	N (%)	N (%)	N (%)	
Sex				
Male	308 (78.6)	196 (75.4)	112 (84.9)	0.031
Female	84 (21.4)	64 (24.6)	20 (15.2)	
SES				
1 (least deprived)	68 (18.4)	48 (19.4)	20 (16.4)	0.013
2	60 (16.3)	50 (20.2)	10 (8.20)	
3	91 (24.7)	59 (23.9)	32 (26.2)	
4	94 (25.5)	53 (21.4)	41 (33.6)	
5 (most deprived)	56 (15.2)	37 (15.0)	19 (15.6)	
Missing	23	13	10	
Comorbidities				
0	57 (14.6)	35 (13.6)	22 (16.8)	
1	111 (28.5)	76 (29.5)	35 (26.7)	0.904
2	94 (24.2)	64 (24.8)	30 (22.9)	
3	60 (15.4)	39 (15.1)	21 (16.0)	
4 or more	67 (17.2)	44 (17.1)	23 (17.6)	
Missing	3	2	1	
Living arrangements				
With family	296 (75.9)	195 (75.2)	101 (77.1)	
Alone	87 (22.3)	60 (23.2)	27 (20.6)	0.755
Other	7 (1.79)	4 (1.54)	3 (2.29)	
Missing	2	1	1	
Mean EQ-5D-3L (SD)	0.79 (0.28)	0.82 (0.25)	0.73 (0.34)	0.002
Mean SAQ-7 (SD)	76.8 (21.1)	77.8 (22.3)	74.9 (20.4)	0.207

SAQ-7, short form Seattle Angina Questionnaire; SES, socioeconomic status.

### Comparing change in PROM scores

**Figure 2:**
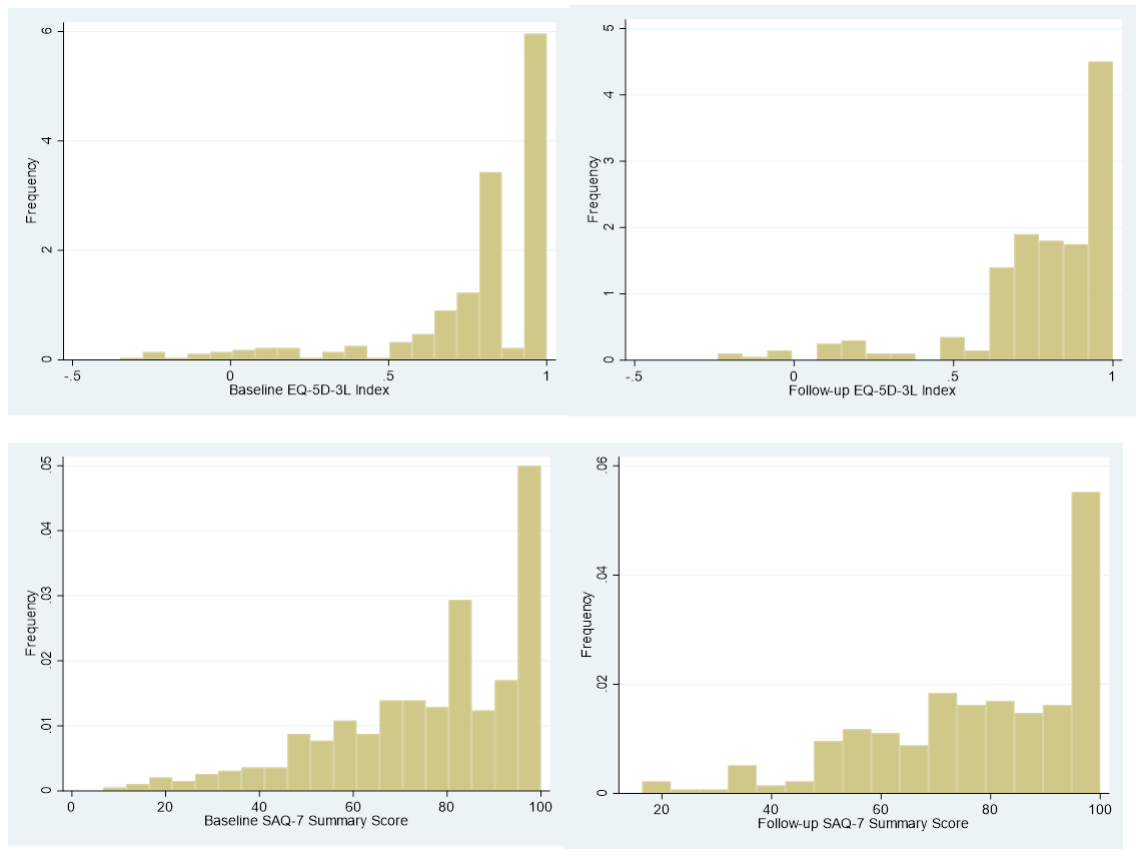
Short form Seattle Angina Questionnaire (SAQ-7) summary and EQ-5D-3L index score distributions.

The distribution of the EQ-5D-3L at baseline has a left skew, with the majority of patients between 0.8 and 1.0 and a small minority having scores below 0.5 indicating poor health-related QoL. The SAQ-7 score distribution at baseline also has a left skew but to a lesser extent than the EQ-5D index (figure 2).

Three months after AMI, patients’ mean SAQ-7 score and mean angina symptom subscale was similar to their baseline score (table 2). In contrast, the physical limitation subscale was worse than at baseline (79.9 vs 73.2, p=0.002) while the SAQ-QoL subscale had improved (66.6 vs 73.9; p<0.001). The EQ-5D-3L index score was slightly lower at 3 months than at baseline (0.82 vs 0.79, p<0.02) though its statistical significance, however, appears to be due to a change in the shape of the distribution rather than a shift in distribution.

**Table 2:** Comparison of baseline and follow-up PROM scores

PROM	Number with complete data	Baseline Mean (SE, 95% CI)	Follow-up Mean (SE, 95% CI)	Change (95% CI, P value)
SAQ-7 summary	259	77.8 (1.27, 75.3 to 80.3)	78.6 (1.22, 76.2 to 81.03)	+0.8 (−1.6 to 3.2, 0.56)
SAQ-7 physical limitation	227	79.9 (1.9, 76.2 to 83.7)	73.2 (1.81, 69.6 to 76.8)	−6.7 (−10.3 to −3.1, 0.0018)
SAQ-7 angina symptom	258	86.9 (1.2 to 84.6)	88.6 (14.1, 86.5 to 90.7)	+1.7 (−13.3 to 16.7, 0.24)
SAQ-QoL	254	66.6 (1.8, 63 to 70.2)	73.9 (1.7, 70.6 to 77.2)	+7.3 (3.9 to 10.7, <0.001)
EQ-5D-3L index	256	0.82 (0.02, 0.79 to 0.85)	0.79 (0.02, 0.76 to 0.82)	−0.03 (−0.07 to 0.01, 0.02)

PROM, patient-reported outcome measure; QoL, quality of life; SAQ-7, short form Seattle Angina Questionnaire.

### Influence of non-response on change in health status

Changes following STEMI and PCI in most PROM scores (the SAQ-7, SAQ-7 subscales and EQ-5D-3L) were not significantly associated with patient characteristics. The one exception was that patients in the poorest health (as determined by their baseline EQ-5D-3L score) reported significantly larger (p<0.001) improvements in their EQ-5D scores at 3 months (table 3).

**Table 3:** Exploring extent of differences in PROM scores and health change with responder characteristics

Patient characteristics	Mean SAQ-7 summary score		SAQ-7 summary score difference in health change, (SD) (n=259)	P value
	Baseline (Q1) score, (SD) (n=390)	Follow-up (QF) score, (SD) (n=260)		
Age				
>70	80.3 (20.5)	78.4 (20.2)	−1.88 (25.0)	0.33
50–70	75.5 (21.0)	78.7 (19.0)	1.72 (22.3)	
<50	75.4 (21.8)	78.9 (20.7)	4.65 (25.5)	
Sex				
Male	77.3 (20.7)	80.4 (19.1)	1.92 (23.2)	0.2
Female	75.2 (22.4)	72.5 (20.7)	−2.46 (24.4)	
SES				
1 (least deprived)	79.6 (20.0)	83.4 (19.0)	5.89 (20.4)	0.23
2	80.0 (18.4)	79.5 (18.6)	−0.96 (23.8)	
3	78.5 (19.0)	77.2 (19.6)	−1.73 (23.0)	
4	72.6 (24.3)	80.4 (21.5)	5.10 (24.5)	
5 (most deprived)	75.1 (20.9)	72.6 (18.8)	−2.85 (27.9)	
EQ-5D baseline categories				
1 (≤0.65)	59.1 (24.4)	64.5 (23.0)	6.3 (33.8)	0.16
2 (0.66–0.85)	74.7 (19.2)	76.4 (18.9)	2.4 (24.1)	
3 (0.86–1)	85.5 (15.9)	84.1 (16.7)	−1.6 (19.6)	

Patient characteristics	EQ-5D			P value*
	Baseline score (n=385)	Follow-up score (n=258)	Difference in health change (n=256)	
Age				
>70	0.80 (0.23)	0.78 (0.26)	−0.01 (0.24)	0.41
50–70	0.80 (0.28)	0.80 (0.22)	−0.05 (0.23)	
<50	0.76 (0.36)	0.74 (0.33)	−0.04 (0.23)	
Sex				
Male	0.80 (0.28)	0.81 (0.24)	−0.04 (0.23)	0.81
Female	0.75 (0.30)	0.71 (0.28)	−0.03 (0.23)	
SES				
1 (least deprived)	0.85 (0.21)	0.81 (0.23)	−0.04 (0.17)	0.38
2	0.83 (0.23)	0.84 (0.19)	−0.01 (0.26)	
3	0.79 (0.23)	0.80 (0.24)	−0.01 (0.23)	
4	0.73 (0.37)	0.78 (0.25)	−0.02 (0.24)	
5 (most deprived)	0.77 (0.34)	0.70 (0.31)	−0.10 (0.22)	
EQ-5D baseline				
Categories				
1 (≤0.65)	0.27 (0.28)	0.49 (0.38)	0.19 (0.33)	<0.001
2 (0.66–0.85)	0.78 (0.06)	0.79 (0.17)	0.01 (0.17)	
3 (0.86–1)	0.98 (0.04)	0.86 (0.19)	−0.12 (0.19)	

*From ANOVA.

PROM, patient-reported outcome measure; SES, socioeconomic status.

### Assessment of non-response bias

Assessment of potential biases that might have been introduced by some patients not responding was based on the assumption that patients with similar baseline EQ-5D index scores would have had similar follow-up EQ-5D or SAQ scores. To illustrate the impact on non-response linked to baseline EQ-5D (mean 0.82 in responders vs 0.79 in non-responders; table 1), we estimated the mean change in SAQ and EQ-5D scores had there been 100% follow-up response rate, compared with the observed mean changes. The mean change in SAQ-7 would have been 1.2 (for all participants including non-responders) compared with 0.8 (observed in responders). The observed mean change in EQ-5D-3L would have been −0.02 compared with the observed mean change of −0.03.

## Discussion

### Main findings

Three-month follow-up PROMs can be successfully collected from two-thirds of patients admitted as emergencies with STEMI for primary PCI through mailed questionnaires. Although responders and non-responders were similar with regards to their living arrangements, number of comorbidities and baseline SAQ-7, responders were more likely to be older, female, of a higher social economic status and be in better general heath according to the EQ-5D-3L score. Apart from the latter, none of these characteristics was associated with the change in health reported at follow-up so have not introduced any possible bias to the findings. However, the higher EQ-5D-3L of responders at baseline could introduce some bias leading to an underestimation of the improvement in the cardiac health of patients 3 months after the event: change in SAQ-7 would be 1.2 instead of 0.8. Similarly, the observed deterioration in generic health (EQ-5D-3L 0.03 lower at follow-up) would be less (0.01 lower).

Three months after PCI, patients’ mean SAQ-7 score and angina symptom score returned to their baseline score, suggesting patients regain their prior level of cardiac health. Although patients reported greater physical limitation than beforehand, they felt their QoL had improved. Given that a clinically meaningful difference in SAQ scores is estimated to be 5–8 points, these differences are clinically important.^24^

Although the EQ-5D-3L index score was lower at 3 months when compared with baseline (0.82 vs 0.79), the clinical significance of this decrement should be further explored as although this reached statistical significance, the CIs overlap.

### What this study adds

This study has demonstrated the feasibility of collecting PROMs 3 months after STEMI among patients who, during their admission, had supplied retrospective accounts of the pre-event health status. It has shown that the response is subject to responder bias which, if confirmed in a larger study, would need to be taken into account when comparing the outcomes of different providers to ensure meaningful findings.

The observation that while patients’ physical limitation worsens their QoL improves is surprising. There are three possible explanations. First, it may be that patients recall their prior QoL as worse than it was, although no such bias was detected in studies of elective surgery when retrospective and contemporary reports were compared.^14 15^ Second, it may be that patients’ baseline disease-specific QoL was already lowered due to the presence of subacute symptoms prior to their AMI, but were not at the clinical threshold that warranted medical attention. Grodzinsky *et al* reported similar baseline SAQ-QoL scores (63.8) in patients with AMI as that reported in this study.^25^

Third, it may be that patients exercised caution in their physical exertion and hence imposed greater physical limitations on their function than necessary. Meanwhile, their QoL may improve from the psychological boost of having survived their AMI and had the reassurance of having had their coronary arteries stented. This may be due to a degree of ‘response shift’ occurring following patients’ experience of an AMI. Patients may have a different appreciation of their cardiac-related QoL. All PROMs, which are subjective reports, can be influenced by response shift.^26^ The literature on clinical recovery trajectories after STEMI at 3 months is sparse with no studies reporting the SAQ-7. However, it is reported that patients continue to recover and improve their SAQ scores for up to 12 months.^9 25^

### Strengths and limitations

This is the first study of using retrospective PROMs in routine clinical practice to collect patients’ baseline health status and 3 months after for those admitted with STEMI in England. Conducting it in five trusts demonstrated the feasibility of PROM use in different hospital organisational cultures and environments.

One potential limitation is that despite the left skew of the EQ-5D data, we opted to use the same statistical test (t-test) for comparisons between the 3 months and baseline to preserve consistency comparisons with other measures. However, as the t-test does not take into account the skew and truncation of the EQ-5D data, the p value should be interpreted with caution in this instance and the CI is the more appropriate method of interpretation of any differences.

### Implications on further research/policy

This study shows that it is feasible to collect retrospective and follow-up PROMs from patients admitted as emergencies with STEMI in NHS hospitals. This approach offers an insight into the opportunity for assessing, from the patient’s perspective, the impact of treatment for the 40% of hospital admissions that are emergencies and patients’ subsequent recovery after their emergency admission. The generalisability of these findings to other causes of emergency admissions needs to be established.

Further research is warranted to explore longer-term outcomes and compare these with patient risk profiles and clinical characteristics to recovery trajectories.

Routine collection of PROMs in emergency admissions is feasible using the retrospective PROMs collected during the index admission and a subsequent contemporary follow-up. Data could be linked to clinical measures known to be associated with outcome (such as Kilip classification, concentration of Tnl, infarct site and left ventricular ejection fraction) and quality dashboards to support on-going quality improvement through benchmarking, by promoting clinical effectiveness and patient-centred care. Larger studies are needed to collect PROMs in patients admitted with AMI and other patients with emergency acute coronary syndrome to enable subgroup analysis of patient and clinical characteristics, to investigate further any response bias and to develop risk adjustment models to enable comparisons of providers.
