# Supplementary material for: Using patient-reported outcome measures for primary percutaneous coronary intervention
Source: Open Heart. 2019 Feb 16;6(1):e000920. doi: 10.1136/openhrt-2018-000920 (PMC6443122; doi:10.1136/openhrt-2018-000920)
Supplement: Supplementary data [file openhrt-2018-000920supp001.docx]

Appendix: Study Flow Diagram

STEMI patients meeting MINAP criteria

and discharged alive

(n= 636)

Patients ineligible
(n =90)

- 47 lacked capacity
- 43 language/ social reasons

Patients participating
(n = 396)

Patients Invited
(n =432)

Patients declined

(n=36)

Patients eligible
(n =547)

Patients not invited
(n =115)
